# Detection of Genomic Regions with Pleiotropic Effects for Growth and Carcass Quality Traits in the Rubia Gallega Cattle Breed

**DOI:** 10.3390/ani11061682

**Published:** 2021-06-04

**Authors:** Maria Martinez-Castillero, Carlos Then, Juan Altarriba, Houssemeddine Srihi, David López-Carbonell, Clara Díaz, Paulino Martinez, Miguel Hermida, Luis Varona

**Affiliations:** 1 Instituto Agroalimentario de Aragón (IA2), Universidad de Zaragoza, 50013 Zaragoza, Spain; carlosmirokyst@gmail.com (C.T.); altarrib@unizar.es (J.A.); houssemsrihi60@gmail.com (H.S.); 767339@unizar.es (D.L.-C.); lvarona@unizar.es (L.V.); 2 Instituto Nacional de Investigación y Tecnología Agraria (INIA), 28040 Madrid, Spain; cdiaz@inia.es; 3 Facultad de Veterinaria, Universidad de Santiago de Compostela, 27002 Lugo, Spain; paulino.martinez@usc.es (P.M.); miguel.hermida@usc.es (M.H.)

**Keywords:** beef cattle, single-step GBLUP, SNP, candidate genes, GWAS, pleiotropy

## Abstract

**Simple Summary:**

The breeding scheme in the Rubia Gallega cattle population is based upon traits measured in farms and slaughterhouses. We have developed a ssGWAS by backsolving the SNP effects after implementing a ssGBLUP. The results showed an apparent heterogeneity of the additive genetic variance across the genome. Some of the genomic regions explaining the most of this additive variance were shared across traits, indicating the presence of pleiotropic effects, which were reflected in their genetic correlations.

**Abstract:**

The breeding scheme in the Rubia Gallega cattle population is based upon traits measured in farms and slaughterhouses. In recent years, genomic evaluation has been implemented by using a ssGBLUP (single-step Genomic Best Linear Unbiased Prediction). This procedure can reparameterized to perform ssGWAS (single-step Genome Wide Association Studies) by backsolving the SNP (single nucleotide polymorphisms) effects. Therefore, the objective of this study was to identify genomic regions associated with the genetic variability in growth and carcass quality traits. We implemented a ssGBLUP by using a database that included records for Birth Weight (BW-327,350 records-), Weaning Weight (WW-83,818-), Cold Carcass Weight (CCW-91,621-), Fatness (FAT-91,475-) and Conformation (CON-91,609-). The pedigree included 464,373 individuals, 2449 of which were genotyped. After a process of filtering, we ended up using 43,211 SNP markers. We used the GBLUP and SNPBLUP model equivalences to obtain the effects of the SNPs and then calculated the percentage of variance explained by the regions of the genome between 1 Mb. We identified 7 regions of the genome for CCW; 8 regions for BW, WW, FAT and 9 regions for CON, which explained the percentage of variance above 0.5%. Furthermore, a number of the genome regions had pleiotropic effects, located at: BTA1 (131–132 Mb), BTA2 (1–11 Mb), BTA3 (32–33 Mb), BTA6 (36–38 Mb), BTA16 (24–26 Mb), and BTA 21 (56–57 Mb). These regions contain, amongst others, the following candidate genes: *NCK1*, *MSTN*, *KCNA3*, *LCORL*, *NCAPG*, and *RIN3*.

## 1. Introduction

The Rubia Gallega is one of the most important breeds of local cattle in Spain’s specialized in meat production, renowned for its meat quality. Currently, the census of the population stands at around 40,000 individuals, and its herd book was established in the 1960s. The Rubia Gallega breeding program started in 1990. The average performance of the breed implies a good growth rate (1200) and low feed conversion rate (~5), with a 60% carcass yield and a calving interval slightly higher than 400 days [1,2]. Morphologically, the Rubia Gallega breed has evolved to a medium-equilibrated morphological type, slightly lower in longitude but with a broader thoracic capacity [3]. The objectives of the breeding program include improving average daily gain and the carcass conformation. Therefore, the criteria of selection of the breeding scheme include traits measured in farms (birth weight (BW) and weaning weight (WW)) and slaughterhouses (cold carcass weight (CCW), conformation (CON), and fatness (FAT)).

Since 2018, genomic selection (GS) under a single-step approach or ssGBLUP [4] has been implemented in numerous breeding programs. In this approach, each SNP marker is provided with the same prior weight. However, some authors have noted that the predictive ability must be improved by providing a greater weight to the SNP markers located within the genomic regions associated with the additive genetic variation in the traits [5].

In this sense, Wang et al. [6] developed a procedure that computes the SNP marker effects and their corresponding p-values by transforming the additive genetic models’ predictions. However, the use of the p-value is a controversy; those obtained by contiguous or very close-by SNPs have a very similar magnitude as a consequence of linkage disequilibrium (LD) in the SNP markers. The regions with a high degree of LD between the markers dilute the association with the analyzed traits, and thus present smaller p-values, even if implemented in a relatively high additive genetic variability of the trait [7]. To solve this problem, some authors have proposed using the percentage of additive genetic variability captured by a group of consecutive markers instead of p-values [4,5].

This study aims to identify genomic regions associated with the genetic variability in growth and carcass quality traits in the Rubia Gallega breed by carrying out GWAS analyses based on predictions obtained by ssGBLUP. Following this procedure, the other objectives of this study were: (1) to identify candidate genes that could contain causal mutations, explaining the important percentage of the genetic variability of the traits and (2) to study the degree of similitude between the distribution of the additive genetic variance across the analyzed traits to characterize pleiotropic regions between them.

## 2. Materials and Methods

### 2.1. Data

The datasets used in the study contained phenotypic information and pedigree, collected by ACRUGA (Asociacion Nacional de Criadores de Ganado Vacuno Selecto de Raza Rubia Gallega). The phenotypic data included 327,350 records for the BW, 83,818 for WW, 91,621 for CCW, 91,609 for CON and 91,475 for FAT (see Table 1). CON was described using the SEUROP scale [8], and converted into a numeric scale from 1 (P- carcass with worst characteristics) to 6 (S-carcass with great characteristics). FAT was described following the 225/08 Real Decreto [9], and it was scaled from 1 (low fat percentage) to 5 (high fat percentage). The pedigree included 464,373 individual dam-sire entries.

We used the Axiom Bovine platform from ThermoFisher Scientific, Waltham, MA, USA to genotype 2455 individuals. Among them, 688 were genotyped with the Axiom_BovMDv2 and 1767 with the Axiom_BovMDv3. The files were merged using PLINK v1.19 [10]. We carried out a standard SNP quality control by setting the number of missing genotypes per individual to less than 95%, resulting in 2449 individuals. Additionally, we excluded SNPs with missing genotypes greater than 5% and minor allele frequency (MAF) lower than 0.01. We only selected the SNP markers located within the autosomal chromosomes, resulting in a total of 43,211 SNPs.

### 2.2. Statistical Models

The model of analysis for traits BW, CCW, CON, and FAT was the following

(1) y=Xb+Vu+Wp+e

where y is the vector of phenotypic observations for the corresponding trait, b is the vector of systematic effects including: the age of the animal, in days, as a covariate (for traits CCW, CON, and FAT); the age of the mother at birth, split into six categories (<2, 3, 4, 5, 6 and >6 years); sex (1-male, and 2-female); slaughterhouse effect, split into 32 categories (for traits CCW, CON and FAT). u is the vector of additive genetic effect, p is the vector of the random effect associated with the herd-year-season (a combination of the herd and the year and season of calving: season 1, calvings from January to March; season 2, calvings from April to June; season 3, calvings from July to September; season 4, calvings from October to December), and e is the vector of residual effects. X, V, and W are the incidence matrices corresponding to the vectors of systematic, additive genetic and herd-year-season effects. The variances of random effects were

(2) $$\mathrm{var}\left[\begin{array}{c} u\\ p\\ e \end{array}\right]=\left[\begin{array}{ccc} H\sigma_{a}^{2} & 0 & 0\\ 0 & I\sigma_{p}^{2} & 0\\ 0 & 0 & I\sigma_{e}^{2} \end{array}\right]$$

where $\sigma_{a}^{2}$ is the additive genetic variance, $\sigma_{p}^{2}$ is the variance associated with herd–year–season, and $\sigma_{e}^{2}$ is the residual variance. Furthermore, the model implemented for the trait WW was the following

(3) y=Xb+Zu+Tm+Wp+e

where m corresponds to the additive genetic maternal effects and T to the corresponding incidence matrix. The systematic effects (**b**) included in the model were: the age of the animal, in days, as a covariate; the age of the mother at birth, split into six categories (<2, 3, 4, 5, 6 and >6 years) and sex (1-male, and 2-female). The variances of the random effects were

(4) $$\mathrm{var}\left[\begin{array}{c} u\\ m\\ p\\ e \end{array}\right]=\left[\begin{array}{cccc} H\sigma_{a}^{2} & H\sigma_{am} & 0 & 0\\ H\sigma_{am} & H\sigma_{m}^{2} & 0 & 0\\ 0 & 0 & I\sigma_{p}^{2} & 0\\ 0 & 0 & 0 & I\sigma_{e}^{2} \end{array}\right]$$

where $\sigma_{m}^{2}$ is the additive genetic maternal variance and $\sigma_{am}$ is the covariance between the additive genetic and the additive genetic maternal variances.

For all models and traits, H is the matrix that combines the numerator relationship matrix (A) with the genomic relationship matrix (G), as described by Aguilar et al. (2010) and I is the identity matrix. The inverse of the H matrix is

(5) $$H^{-1\;}=A^{-1}+\left[\begin{array}{cc} 0 & 0\\ 0 & G^{-1}-A_{22}^{-1} \end{array}\right]$$

where A is the relationship matrix corresponding to all the individuals, $A_{22}$ is the relationship matrix corresponding to the genotyped individuals, and G is the genomic relationship matrix, obtained following the procedure described by VanRaden [11] as

(6) $$G=\frac{ZZ^{\prime }}{\sum_{i=1}^{N}2{\hat{p}}_{i}\left(1-{\hat{p}}_{i}\right)}$$

where $Z^{\prime }$ is the matrix which contains the genes and adjusted for the estimated allelic frequencies for each of the SNPs in the population ($p_{i}$), and N is the number of SNPs. Additionally, we estimated the SNP effects (g) following the criterion by Wang et al [6]

(7) $$\hat{g}=\mathrm{cov}\left(g,u^{\prime }\right)\left[\mathrm{var}\left(u\right)\right]\hat{u}$$

(8) $$\hat{g}=\frac{Z^{\prime }G^{-1}\hat{u}}{\sum_{i=1}^{N}2{\hat{p}}_{i}\left(1-{\hat{p}}_{i}\right)}$$

(9) $$\hat{g}=Z^{\prime }{\left(Z^{\prime }Z\right)}^{-1}\hat{u}$$

which we later used to estimate the variance explained by the SNP effect [12]:

(10) $${\hat{\sigma }}_{u,i}^{2}={\hat{u}}_{i}^{2}2{\hat{p}}_{i}\left(1-{\hat{p}}_{i}\right)$$

Firstly, we estimated the variance components by the restricted maximum likelihood (REML) [13], using the software AIREMLF90—from the family of software programs BLUPF90 [14]—discarding the first rounds of iterations with the option “EM-REML 100”. We then carried out the GWAS analysis by the software program POSTGSF90 (Athens, GA, USA) [15], also from the family of software programs BLUPF90 [14], with the added option ‘windows_variance 1’ to calculate the proportion of the additive genetic variance associated with the region of the genome determined by a distance of pair bases of 1 Mb. We selected the genome regions that explained an additive genetic variance above 0.5%. We used the BiomartTool (www.ensembl.org, Accessed on 25 March 2021), which contains the latest version of the bovine genome, *Bos Taurus* (ARS-UCD1.2), to identify the genes present in those regions.

## 3. Results

### 3.1. Genetic Parameters

The estimates of variance components are shown in Table 2. The lowest variation was due to the herd–year–season effect for carcass quality traits (CCW, FAT, CON), the genetic effect for BW and the maternal effect for WW. The highest variation was due to the residual for traits BW and FAT, and due to the genetic effect for WW, CCW, FAT, and CON. The heritability estimates, also shown in Table 2, ranged from moderate to high, ranging from 0.230 (BW) to 0.641 (CCW), and were highest for carcass quality traits (CCW, FAT and CON) compared to growth traits (BW and WW). The maternal genetic heritability for trait WW was low (0.135), and the correlation between the genetic and maternal heritabilities was negative and high (−0.736).

### 3.2. Genome-Wide Association Studies (GWAS)

**Figure 1:**
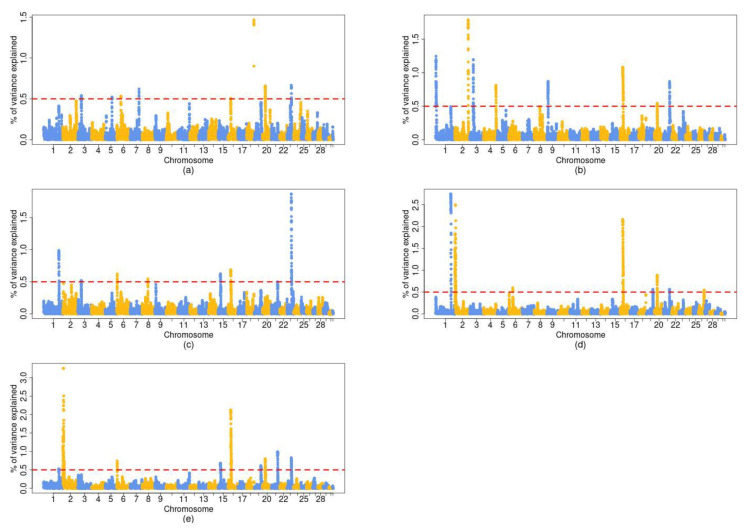
Manhattan plot of genomic sweep from the standardized additive genetic variance (y axis) explained at each of the SNPs by a distance of 1 Mb of pair bases, for traits: (**a**) BW: Birth Weight, (**b**) WW: Weaning Weight, (**c**) CCW: Cold Carcass Weight, (**d**) FAT: Fatness, and (**e**) CON: Conformation with the identified genomic regions that explained the percentage of additive genetic variance above 0.5% (dashed red line).

The results on the GWAS for each of the traits are shown in Figure 1. The figure represents a Manhattan plot of the genomic sweep from the standardized additive genetic variance, explained at each of the SNPs, determined by a distance of pair bases of 1 Mb. The regions which explained an additive genetic variance (Figure 1) above 0.5% were 8 for trait BW, 8 for trait WW, 7 for trait CCW, 9 for trait CON, and 8 for trait FAT. From all the genomic regions and traits, we found 9 regions with pleiotropic effects (Table 3), such as the ones located at BTA1 (131–132 Mb) for WW, CCW, FAT and CON, BTA2 (1–11) for FAT and CON, and BTA16 (24–26) for all traits (BW, WW, CCW, CON, FAT), amongst others.

## 4. Discussion

The large proportion of the phenotypic variance explained by additive genetic effects defines the moderate to high heritabilities obtained. The estimates of heritabilities are in accordance with the estimates obtained for other populations [16,17,18]. The maternal and additive genetic effects obtained for trait WW are similar to those obtained in other studies [19,20]. Furthermore, the correlation between the additive genetic and additive maternal genetic was to the same degree as those obtained in the study by Varona et al [21].

The results acquired from the ssGWAS show that the additive genetic variance obtained is not homogeneously distributed along the genome. Moreover, some regions explain a greater proportion compared to others. The presence of pleiotropy was confirmed when we analyzed the genomic regions of the genome accounting for the percentage of additive genetic variance above 0.5% (see Table 3). Furthermore, the presence of pleiotropy is reflected in the genetic correlation between the traits, as observed in other studies [18,22].

There are two genomic regions, located on chromosomes BTA2 and BTA6, that were highlighted in previous studies [23] as susceptible to host genes related to growth and carcass quality traits. The first region (between 189886 and 9845870 base pairs) contains the *Myostatin* (*MSTN*) gene. The *MTSN* gene is a growth differentiation factor associated with beef cattle’s double-muscled phenotype [24], causing calving difficulty [25]. This study showed a strong association with the genetic variability in traits CON and FAT, which corroborates previous studies [23,26]. Previous studies have confirmed that some of the mutations found in the *MTSN* gene [27] are segregated in the population [28]. However, the length of the genomic region could indicate other genes associated with the existing genetic variability of the traits of interest in this study [29].

The second featured genomic region is on chromosome BTA6, between 36986502 and 37463048 base pairs. In this region, there are a number of candidate genes such as *Leucine Aminopeptidase 3* (*LAP3*), *FAM184B*, *DDB1* and *CUL4 associated factor 16* (*DCAF16*), *Ligand Dependent Nuclear Receptor Corepressor Like* (*LCORL*), and *Non-SMC Condensin I Complex Subunit G* (*NCAPG*). The *DCAF16* mediates the processes of ubiquitination and proteasome-dependent degradation of nuclear proteins [30,31], and *NCAPG* is a gene involved in cell division and mitotic chromosome condensation [32]. The *DCAF16*-*NCAPG* regions have been associated with average daily gain in beef cattle [33]. *NCAPG* has been associated with postnatal growth and cold carcass weight in cattle [34,35], fat deposition [36,37], and carcass and meat quality [6,38]. Furthermore, *LAP3* and *FAM184B* have been associated with growth and carcass traits [39,40].

Further on, on chromosome BTA1 (between 131853658 and 132433521 base pairs), we identified the candidate gene *Cytoplasmic protein* (*NCK1*). *NCK1* is a protein-coding gene located in the cytoplasm and involved in translation regulation processes [41], associated with growth traits in beef cattle [42]. On chromosome BTA3, between 32316119 and 32766506 base pairs, we identified the candidate gene *Potassium Voltage-Gated Channel Subfamily A Member 3* (*KCNA3*), which encodes a member of the voltage-gated potassium channel involved in the proliferation and activation of the T-cells [43]; it is associated with the marbling of meat in Korean beef cattle [44]. On chromosome BTA16, between 24983313 and 25935379 base pairs, we identified the candidate gene *Dual Specifity Phosphatase 10* (*DUSP10*), a dual specificity protein that inhibits members of the MAP (Mitogen-activated protein) kinase family, associated with the proliferation and differentiation of cells [45]. *DUSP10* is associated with beef cattle’s growth traits, as average daily gain and carcass weight [46,47]. Finally, on chromosome BTA21, between 56755228 and 57504101 base pairs, we identified the candidate genes *Ras and Rab Interactor 3* (*RIN3*) and *Legumain* (*LGMN*). *RIN3* is a Ras interaction-interference effector protein that binds to RAB5 to exchange GDP (Guanosine triphosphate) for free GTP (Guanosine diphosphate) [48,49], and it is found to be associated with growth traits [50]. *LGMN* is a protein-coding gene for a cysteine protease with specificity for hydrolysis of asparaginyl bonds, involved in regulating the processing of MHC class II antigen proteins in the lysosomal or endosomal systems [51]. Moreover, *LGMN* is associated with adipose tissue expression changes, significant for meat quality in beef cattle [52]. However, no candidate genes associated with beef cattle were found on chromosomes 15, 20 and 23.

From the results we obtained, we observed heterogeneity in the additive genetic variability across the genome. These results could serve as the basis for the modification of future procedures of genomic assessment in the Rubia Gallega population. At present, the implementation of genomic assessment is in the initial phases, and it uses the GBLUP procedure, following the procedure by VanRaden [12]. However, this procedure assumes that all the SNP markers included in the analysis are related in the same way as the analyzed trait’s genetic variability. However, other studies have observed that some genomic regions are more involved than others in the regulation of the traits of interest [53,54,55].

Furthermore, the GBLUP procedure weights each SNP marker’s information to calculate the genomic relationship matrix [5]. Nonetheless, this consideration depends on the traits and hinders the implementation of multi-trait analysis in the genomic assessment. A multi-trait assessment could provide a relevant increase in precision, especially for those traits that are obtained post-mortem (CCW, CON, and FAT). Future studies should assess the implementation of procedures that allow a ponderation of the SNP markers for each trait, and should be compatible with the multivariate studies, such as the one recently proposed by the authors of [56]. 

## 5. Conclusions

The results of this study confirm the moderate to high heritability of growth and carcass quality traits in beef cattle populations. They also show that the additive genetic variation is heterogeneously distributed across the genome. Finally, it was observed that the genomic regions explaining most of the additive genetic variance are shared among the traits due to pleiotropy.

## Figures and Tables

**Table 1:** Number of records, phenotypic means, and standard deviation of growth and carcass quality traits.

Trait	Records	Mean	SD
Birth Weight, kg (BW)	327,350	42.60	7.24
Weaning Weight, kg (WW)	83,818	284.63	47.49
Cold Carcass Weight, kg (CCW)	91,621	223.47	40.38
Fatness, % (FAT) ^1^	91,475	2.27	0.44
Conformation (CON) ^2^	91,609	3.95	0.66

^1^ Fatness, % (FAT): measured following the 225/08 Real Decreto, scaled from 1 to 5. ^2^ Conformation (CON): measured using the SEUROP scale, converted to numeric from 1 (P) to 6 (S).

**Table 2:** Estimates of variance components (standard deviation between brackets), heritabilities and correlations ^1^ of growth and carcass quality traits ^2^.

	BW	WW	CCW	FAT	CON
$\sigma_{p}^{2}$	12.70 (0.12)	398.04 (7.75)	182.27 (3.83)	0.012 (0.5 × 10^−3^)	0.033 (0.1 × 10^−2^)
$\sigma_{a}^{2}$	10.89 (0.19)	894.79 (54.48)	724.43 (15.89)	0.078 (0.2 × 10^−2^)	0.219 (0.5 × 10^−2^)
$\sigma_{\mathrm{mat}}^{2}$	-	301.97 (21.96)	-	-	-
$\sigma_{a\_\mathrm{mat}}$	-	−382.72 (30.85)	-	-	-
$\sigma_{e}^{2}$	23.68 (0.13)	639.95 (26.36)	224.16 (10.04)	0.098 (0.2 × 10^−2^)	0.136 (0.3 × 10^−2^)
$h^{2}$	0.230	0.400	0.641	0.415	0.565
$h_{\mathrm{mat}}^{2}$	-	0.135	-	-	-
$r_{a\_\mathrm{mat}}$	-	−0.736	-	-	-

^1^ $\sigma_{p}^{2}$: herd–year–season variance; $\sigma_{a}^{2}$: animal additive genetic variance; $\sigma_{\mathrm{mat}}^{2}$: maternal additive genetic variance; $\sigma_{a_{\_\mathrm{mat}}}$: covariance between the additive genetic and maternal additive genetic effects; $\sigma_{e}^{2}$: residual variance; $h^{2}$: heritability; $h_{\mathrm{mat}}^{2}$: maternal heritability; $r_{a_{\_\mathrm{mat}}}$ correlation between the genetic and maternal heritabilities. ^2^ Traits: Birth Weight (BW), Weaning Weight (WW), Cold Carcass Weight (CCW), Fatness (FAT), and Conformation (CON).

**Table 3:** Regions of the genome that explained a percentage of variance above 0.5% and the pair bases distance, the traits ^1^ in which these regions appear, and the candidate genes with a pleiotropic effect.

Chromosome	BP ^2^	BW	WW	CCW	FAT	CON	CG ^3^
1	131–132	-	X	X	X	X	*NCK1*
2	1–11	-	-	-	X	X	*MSTN*
3	32–33	X	X	X	-	-	*KCNA3*
6	36–38	X	-	X	X	X	*DCAF16*, *LCORL*, *NCAPG*, *LAP3*, *FAM184B*
15	23–24	-	-	X	-	X	-
16	25–26	X	X	X	X	X	*DUSP10*
20	21–22	X	X	-	X	X	-
21	56–57	-	X	-	X	X	*RIN3*, *LGMN*
23	38–39	X	-	X	-	X	-

^1^ Traits: Birth Weight (BW), Weaning Weight (WW), Cold Carcass Weight (CCW), Fatness (FAT), and Conformation (CON). ^2^ BP: distance, in Mb. ^3^ CG: candidate genes; *NCK1* = Cytoplasmic protein; *MSTN* = Myostatin; *KCNA3* = Potassium Voltage-Gated Channel Subfamily A Member 3; *DCAF16* = DDB1 and CUL4 associated factor 16, *LCORL* = Ligand Dependent Nuclear Receptor Corepressor Like; *NCAPG* = Non-SMC Condensin I Complex Subunit G; *LAP3* = Leucine Aminopeptidase 3; *DUSP10* = Dual Specifity Phosphatase 10; *RIN3* = Ras and Rab Interactor 3; *LGMN* = Legumain.

## Data Availability

Data will be available under reasonable request to L.V.
